# LncRNAs GIHCG and SPINT1-AS1 Are Crucial Factors for Pan-Cancer Cells Sensitivity to Lapatinib

**DOI:** 10.3389/fgene.2019.00025

**Published:** 2019-02-19

**Authors:** Zhen Xiang, Shuzheng Song, Zhenggang Zhu, Wenhong Sun, Jaron E. Gifts, Sam Sun, Qiushi Shauna Li, Yingyan Yu, Keqin Kathy Li

**Affiliations:** ^1^Department of Surgery of Ruijin Hospital, and Shanghai Institute of Digestive Surgery, Shanghai Key Laboratory for Gastric Neoplasms, Shanghai Jiao Tong University School of Medicine, Shanghai, China; ^2^Guangxi Key Laboratory of Processing for Non-ferrous Metal and Featured Materials, Research Center for Optoelectronic Materials and Devices, School of Physical Science Technology, Guangxi University, Nanning, China; ^3^Department of Chemistry, Center for Diagnostics and Therapeutics, Georgia State University, Atlanta, GA, United States

**Keywords:** pan-cancer, computational analysis, LncRNAs, lapatinib, targeted therapy

## Abstract

Lapatinib is a small molecule inhibitor of EGFR (HER1) and ERBB2 (HER2) receptors, which is used for treatment of advanced or metastatic breast cancer. To find the drug resistance mechanisms of treatment for EGFR/ERBB2 positive tumors, we analyzed the possible effects of lncRNAs. In this study, using CCLE (Cancer Cell Line Encyclopedia) database, we explored the relationship between the lncRNAs and Lapatinib sensitivity/resistance, and then validated those findings through *in vitro* experiments. We found that the expression of EGFR/ERBB2 and activation of ERBB pathway was significantly related to Lapatinib sensitivity. GO (Gene Oncology) analysis of top 10 pathways showed that the sensitivity of Lapatinib was positively correlated with cell keratin, epithelial differentiation, and cell-cell junction, while negatively correlated with signatures of extracellular matrix. Forty-four differentially expressed lncRNAs were found between the Lapatinib sensitive and resistant groups (fold-change > 1.5, *P* < 0.01). Gene set variation analysis (GSVA) was performed based on 44 lncRNAs and genes in the top 10 pathways. Five lncRNAs were identified as hub molecules. Co-expression network was constructed by more than five lncRNAs and 199 genes in the top 10 pathways, and three lncRNAs (GIHCG, SPINT1-AS1, and MAGI2-AS3) and 47 genes were identified as close-related molecules. The three lncRNAs in epithelium-derived cancers were differentially expressed between sensitive and resistant groups, but no significance was found in non-epithelium-derived cancer cells. Correlation analysis showed that SPINT1-AS1 (*R* = −0.715, *P* < 0.001) and GIHCG (*R* = 0.557, *P* = 0.013) were correlated with the IC50 of epithelium-derived cancer cells. In further experiments, GIHCG knockdown enhanced cancer cell susceptibility to Lapatinib, while high level of SPINT1-AS1 was a sensitive biomarker of NCI-N87 and MCF7 cancer cells to Lapatinib. In conclusions, lncRNAs GIHCG and SPINT1-AS1 were involved in regulating Lapatinib sensitivity. Up-regulation of GIHCG was a drug-resistant biomarker, while up-regulation of SPINT1-AS1 was a sensitive indicator.

## Introduction

Lapatinib is a small molecular drug that has been shown to be a dual tyrosine kinase inhibitor, which is involved in the EGFR/HER1 and ERBB2/HER2 pathways and suppresses the autophosphorylation of these receptors. Clinically, it has been used in combination therapy with capecitabine in patients with advanced or metastatic breast cancer that overexpressed ERBB2/HER2 in the cases of previous treatment with anthracyclines, taxanes, or trastuzumab (Herceptin) (Geyer et al., 2006). In addition, a satisfactory response rate has also been found with Lapatinib treatment for ERBB2-positive progressive gastric cancer (Cetin et al., 2014; Satoh et al., 2014). However, in patients with head and neck squamous cell carcinoma, Lapatinib combined with radiotherapy did not show therapeutic effects (Harrington et al., 2015). Similarly, in ERBB2/EGFR positive metastatic bladder cancer patients who underwent first-line chemotherapy didn't get benefit from Lapatinib maintenance treatment (Powles et al., 2017). Therefore, uncovering the drug-resistant mechanism of Lapatinib will help improve the therapeutic effects of Lapatinib targeted therapy and find new sensitive biomarkers.

Long non-coding RNAs (lncRNAs) are a large class of transcribed RNA molecules that are longer than 200 nucleotides but do not encode proteins. In addition to the regulation of diverse cellular processes, such as epigenetics, cell cycle, and cell differentiation, they have been found to play important roles in carcinogenesis, tumor development, and treatment resistance (Heery et al., 2017; Peng et al., 2017; Hahne and Valeri, 2018; Wang et al., 2018; Wu et al., 2018). For instance, Ma et al. found that lncRNAs CASC9 and EWAST1 were two crucial molecules associated to EGFR-TKIs resistant in non-small cell lung cancer (Ma et al., 2017).

The Cancer Cell Line Encyclopedia (CCLE) database (https://portals.broadinstitute.org/ccle) is an open access resource with the most completely integrated datasets of cancer cells genomes and drug effectiveness. It includes the experimental datasets of drug treatment of 24 kinds of chemical compounds in almost 1,000 cancer cell lines of various human cancers (Barretina et al., 2012). Kim et al. used CCLE database in their recent publication. They found that high levels of FGFR and integrin β3 are resistant to crizotinib treatment, suggesting that FGFR, and integrin β3 could be predictive markers for Met-targeted therapy (Kim et al., 2015). To date, there is a limited number of studies (Jiang et al., 2014; Niknafs et al., 2016; Bester et al., 2018; Li D. et al., 2018; Sun et al., 2018) to explore lncRNAs by CCLE database. In this study, we analyzed the lncRNAs of whole-genome datasets of CCLE after treatment with Lapatinib on pan-cancer cell lines, and proposed crucial lncRNAs GIHCG and SPINT1-AS1 involved in regulating Lapatinib sensitivity.

## Materials and Methods

### Data Extraction From CCLE

There are 5,344 lncRNA probes and 49,331 non-lncRNA probes in the whole-genome gene expression profile chip used in CCLE (Barretina et al., 2012). There are 1,037 cell lines of various cancer types in the database. Among those, 504 cell lines had been treated with Lapatinib and got IC50 (half maximal inhibitory concentration) data and 501 cell lines were examined by microarrays. Since the study focused on solid tumors, we deleted cell lines of hematopoietic and lymphoid cell lines. Finally, 420 solid tumor cell lines were enrolled in the study (Table 1).

**Table 1:** The distribution of 420 cancer cell lines of solid tumor.

**Cancer types**	**Count**
Autonomic ganglia	10
Biliary tract	1
Bone	11
Breast	29
Central nervous system	29
Endometrium	20
Kidney	9
Large intestine	23
Liver	19
Lung	91
Esophagus	15
Ovary	28
Pancreas	28
Pleura	7
Prostate	3
Salivary gland	1
Skin	40
Soft tissue	12
Stomach	18
Thyroid	5
Upper aerodigestive tract	7
Urinary tract	14

### Cancer Cell Lines and Cell Culture

Nineteen cancer cell lines were used for validating experiments *in vitro*. Four of those were gastric cancer cell lines (NCI-N87, SGC-7901, AGS, and MKN-45), three were melanoma cell lines (MuM-2C, MV3, and A-375), three were hepatocarcinoma cell lines (LM3, 97L, and Huh7), three were thyroid cancer cell lines (KHM-5M, CAL-62, and C643), two were breast cancer cell lines (MCF7 and SK-BR-3), two were pancreatic cancer cell lines (TCC-PAN2 and BxPC3), and two were colorectal cancer lines (DLD-1 and NCIH-747). Cell lines NCI-N87, MuM-2C, LM3, MV3, Huh7, SGC-7901, CAL-62, AGS, MCF7, C643, 97L, SK-BR-3, KHM-5M, A-375, TCC-PAN2, MKN-45, and BxPC3 were purchased from the Cell Bank of Type Culture Collection of Chinese Academy of Sciences (Shanghai, China). Cell lines DLD-1 and NCIH-747 were purchased from The Global Bioresource Center ATCC (Maryland, USA). The cell lines were cultured in RPMI-1640 supplemented with 10% fetal bovine serum in a humidified incubator at 37°C with 95% air and 5% CO2.

### Transient Transfection of siRNAs

SPINT1-AS1 and GIHCG siRNAs were transfected into cancer cells by Lipofectamine 2000 (Invitrogen, Carlsbad, California, USA) according to the manufacturer's instructions. The siRNA sequences are shown in Table S1.

### RNA Extraction and Quantitative Real-Time PCR Analysis

Total RNA was isolated using the TRIzol solution (Invitrogen, California, USA). The cDNA was synthesized using Reverse Transcription kit (TOYOBO, Japan). Real-time PCR was performed in 10 μl reaction mixtures with the HT 7900 (Applied Biosystems, Foster City, USA) using SYBR™ Select Master Mix (Applied Biosystems, Foster City, USA). The sequences of primers were designed and synthesized by Sunny Biotech (Shanghai, China): The primer sequences are shown in Table S1.

### Cell Viability Assay

Five thousand cells of different cancers were placed in each well of 96-well plates (100 μl/well). Different concentrations of Lapatinib (Selleck, Houston, USA) were incubated for 48 h. After adding 10 μl CCK-8 for 2 h, OD value was measured at 450 nm by spectrophotometry (BioTek, Vermont, USA).

### Data Analysis

The “corrplot” and “pheatmap” package in R software were utilized for visualizing pearson correlation analysis and cluster analysis by “euclidean” method. The Benjamini and Hochberg method was used to calculate P. adjust value. By means of “clusterProfiler” package in R, GSEA (Gene Set Enrichment Analysis) and GO (Gene Ontology) analyses were carried out to explore involved gene clusters. GSEA is a computational method based on previous publication by Subramanian et al. (2005). GO analysis is a kind of gene enrichment analysis to classify gene set on three aspects: molecular function, cellular component and biological process (Ashburner et al., 2000). Differentially expressed lncRNAs and genes with difference larger than 1.5-fold were obtained by “limma” package, which is often used to explore differentially expressed genes between two phenotypes (Ritchie et al., 2015). The top 10 gene clusters of all cancer cell lines were scored using “GSVA” package (*G*ene *S*et *V*ariation *A*nalysis,) in R language, which utilizes non-parametric unsupervised method for evaluating gene set enrichment (GSE) in transcriptomic data (Hanzelmann et al., 2013). Cytoscape software was applied to establish co-expression network and determine hub lncRNAs. The inhibiting ratio and Lapatinib IC50 were calculated according to OD value by GraphPad Prism 6.0 (Inc., La Jolla, CA, USA). The relative RNA levels were calculated by 2^−ΔΔCT^ (ΔCT = LncRNA^CTvalue^ − GAPDH^CTvalue^, ΔΔCT = ΔCT−ΔCT^min^, ΔCT^min^: minimum ΔCT of expression levels of lncRNA GIHCG or SPINT1-AS1 in cell line). Student's *t*-tests were performed by GraphPad Prism 6.0. *P* < 0.05 was considered statistically significant.

## Results

### Lapatinib IC50 From Pan-Cancer Cell Lines Analysis

**Figure 1:**
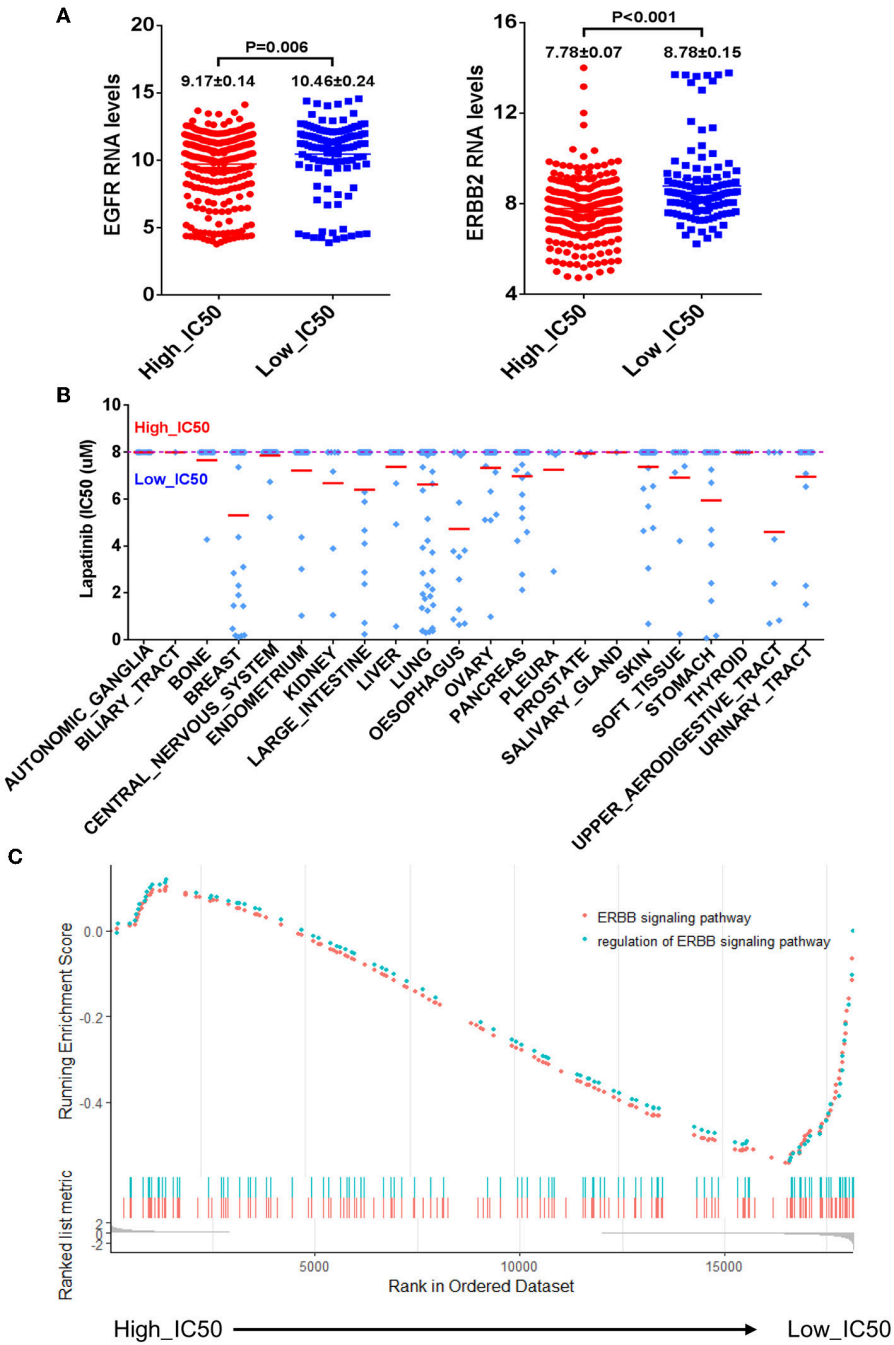
The correlation of mRNA expression levels of EGFR and ERBB2 and Lapatinib IC50. **(A)** The bar charts of mRNA expression levels of EGFR (left) and ERBB2 (right) of cancer cell lines between the high_IC50 and low_IC50 groups of Lapatinib drug. The expression levels of EGFR and ERBB2 are significantly higher in the low_IC50 group than that in the high_IC50 group (*p* < 0.01). **(B)** The distribution tendency of 22 types of solid cancer cell lines in high-IC50 (up to 8 μM) and low_IC50 (lower than 8 μM). The red lines represent mean value of Lapatinib IC50. **(C)** The enrichment analysis of ERBB signaling pathway reveals that ERBB signaling pathway is significantly enriched in Lapatinib low_IC50 group. “Y” axis indicates the enrichment score (ES) value, and “X” axis indicates genes according to differential expression value between high_IC50 and low_IC50 groups. The blue and red dot curves represent ES value. The bottom barcodes represent the leading gene set that strongly contributed to ES value. The positive ES value represents positive correlation to Lapatinib IC50, and minus ES value represents negative correlation to Lapatinib IC50.

The CCLE data of Lapatinib IC50 of the selected 420 cell lines was shown in Table 2. The upper limit of IC50 was originally determined as 8 μM for those cancer cell lines in the database. There were 302 cancer cell lines with IC50 higher than 8 μM, which were insensitive to Lapatinib drug. There were 118 cancer cell lines with IC50 lower than 8 μM, which were relatively sensitive to Lapatinib drug. Taking 8 μM of IC50 as a threshold, we categorized 420 cancer cell lines into two groups, high_IC50 (*n* = 302) and low_IC50 (*n* = 118). Since EGFR and ERBB2 are the targets of the Lapatinib drug, the expression levels of EGFR, and ERBB2 in high_IC50 and low_IC50 groups were analyzed. The expression levels of EGFR and ERBB2 were significantly higher in low-IC50 group than in high_IC50 (Figure 1A, *P* = 0.006 and *P* < 0.001, respectively). The distribution tendency of 22 types of solid cancer cell lines in high-IC50 (up to 8 μM) and low_IC50 (lower than 8 μM) groups is presented in Figure 1B. GSEA analysis showed that ERBB pathway-related genes were enriched in low_IC50 group (Figure 1C, ERBB signaling pathway NES = −1.81, *P* < 0.002, p. adjust = 0.064; regulation of ERBB signaling pathway NES = −1.69, *P* < 0.002, p. adjust = 0.064).

**Table 2:** Lapatinib IC50 of 420 cancer cell lines.

**CCLE cell line names**	**Cell type**	IC50 (μM)^*^
SNU1	Stomach	8
KMRC2	Kidney	8
HEYA8	Ovary	8
NCIH1915	Lung	8
SH10TC	Stomach	8
JMSU1	Urinary tract	8
UACC62	Skin	8
SKLU1	Lung	8
ES2	Ovary	8
SNU398	Liver	8
MSTO211H	Pleura	8
HMC18	Breast	8
HS229T	Lung	8
HS895T	Skin	8
NCIH1092	Lung	8
8505C	Thyroid	8
RKO	Large intestine	8
SW1573	Lung	8
NCIH2172	Lung	8
IGR37	Skin	8
T24	Urinary tract	8
NCIH1581	Lung	8
HLF	Liver	8
MG63	Bone	8
HS840T	Upper aerodigestive tract	8
DMS114	Lung	8
HS936T	Skin	8
FU97	Stomach	8
NCIH2052	Pleura	8
8305C	Thyroid	8
RERFLCAI	Lung	8
SW579	Thyroid	8
TOV112D	Ovary	8
HS729	Soft tissue	8
KMRC1	Kidney	8
SJSA1	Bone	8
HUH1	Liver	8
1321N1	Central nervous system	8
TC71	Bone	8
KELLY	Autonomic ganglia	8
NCIH520	Lung	8
IGR39	Skin	8
EN	Endometrium	8
U118MG	Central nervous system	8
639V	Urinary tract	8
HGC27	Stomach	8
UMUC3	Urinary tract	8
42MGBA	Central nervous system	8
SKNBE2	Autonomic ganglia	8
CALU1	Lung	8
NCIH211	Lung	8
HEC59	Endometrium	8
BFTC909	Kidney	8
RPMI7951	Skin	8
IPC298	Skin	8
NCIH1651	Lung	8
MDAMB436	Breast	8
SKNDZ	Autonomic ganglia	8
DKMG	Central nervous system	8
IALM	Lung	8
NCIH1792	Lung	8
JHH6	Liver	8
PSN1	Pancreas	8
HOS	Bone	8
CAL78	Bone	8
U87MG	Central nervous system	8
GI1	Central nervous system	8
NCIH1155	Lung	8
SBC5	Lung	8
IMR32	Autonomic ganglia	8
NCIH460	Lung	8
WM2664	Skin	8
MEWO	Skin	8
BT549	Breast	8
SKMEL30	Skin	8
NCIH1703	Lung	8
HEP3B217	Liver	8
TT2609C02	Thyroid	8
HEPG2	Liver	8
SKNAS	Autonomic ganglia	8
NCIH1944	Lung	8
SW1271	Lung	8
COLO679	Skin	8
DAOY	Central nervous system	8
SHP77	Lung	8
NCIH1299	Lung	8
VMRCRCZ	Kidney	8
LOXIMVI	Skin	8
NCIH1339	Lung	8
HS746T	Stomach	8
SKHEP1	Liver	8
NCIH1694	Lung	8
COV504	Ovary	8
NCIH1793	Lung	8
SNU423	Liver	8
JHUEM2	Endometrium	8
CALU6	Lung	8
J82	Urinary tract	8
UACC257	Skin	8
G402	Soft tissue	8
MESSA	Soft tissue	8
HT1080	Soft tissue	8
MPP89	Pleura	8
OVTOKO	Ovary	8
SUIT2	Pancreas	8
SIMA	Autonomic ganglia	8
H4	Central nervous system	8
WM1799	Skin	8
A673	Bone	8
NCIH1975	Lung	8
MDAMB157	Breast	8
SKMEL5	Skin	8
SKES1	Bone	8
NCIH2452	Pleura	8
NCIH647	Lung	8
SAOS2	Bone	8
NCIH2023	Lung	8
NCIH226	Lung	8
SF295	Central nervous system	8
SW620	Large intestine	8
NCIH661	Lung	8
HS939T	Skin	8
HS578T	Breast	8
HCC44	Lung	8
EFO21	Ovary	8
KPNSI9S	Autonomic ganglia	8
SF126	Central nervous system	8
HS739T	Breast	8
NCIH1693	Lung	8
TOV21G	Ovary	8
KALS1	Central nervous system	8
A375	Skin	8
CHP212	Autonomic ganglia	8
SW1990	Pancreas	8
LOUNH91	Lung	8
OV90	Ovary	8
SKMEL2	Skin	8
NCIH23	Lung	8
YKG1	Central nervous system	8
WM88	Skin	8
ACHN	Kidney	8
SKNFI	Autonomic ganglia	8
DU145	Prostate	8
GAMG	Central nervous system	8
MDAMB435S	Skin	8
NCIH2087	Lung	8
NCIH1563	Lung	8
HEC6	Endometrium	8
NCIH2228	Lung	8
SW1353	Bone	8
RD	Soft tissue	8
SNU387	Liver	8
OC316	Ovary	8
SKNSH	Autonomic ganglia	8
FUOV1	Ovary	8
LCLC103H	Lung	8
HCC15	Lung	8
KNS60	Central nervous system	8
PK45H	Pancreas	8
HT1197	Urinary tract	8
KP4	Pancreas	8
GB1	Central nervous system	8
HT144	Skin	8
U2OS	Bone	8
HLE	Liver	8
COLO741	Skin	8
TCCSUP	Urinary tract	8
LN18	Central nervous system	8
NCIH810	Lung	8
JHH2	Liver	8
T98G	Central nervous system	8
QGP1	Pancreas	8
IGROV1	Ovary	8
LN229	Central nervous system	8
OVCAR4	Ovary	8
JHH4	Liver	8
HS944T	Skin	8
BCPAP	Thyroid	8
HS683	Central nervous system	8
NCIH2009	Lung	8
GMS10	Central nervous system	8
G401	Soft tissue	8
A172	Central nervous system	8
HEC1B	Endometrium	8
HEC251	Endometrium	8
SW900	Lung	8
OC315	Ovary	8
JHOS2	Ovary	8
RERFLCMS	Lung	8
ISTMES1	Pleura	8
RVH421	Skin	8
MFE296	Endometrium	8
HS766T	Pancreas	8
HCC78	Lung	8
MKN7	Stomach	8
C32	Skin	8
HEC265	Endometrium	8
NCIH1184	Lung	8
SW480	Large intestine	8
NCIH522	Lung	8
NCIH650	Lung	8
OC314	Ovary	8
COV318	Ovary	8
HS852T	Skin	8
NCIH727	Lung	8
EFO27	Ovary	8
SJRH30	Soft tissue	8
KNS81	Central nervous system	8
SNU449	Liver	8
A2058	Skin	8
HS294T	Skin	8
SNU182	Liver	8
COLO205	Large intestine	8
HUCCT1	Biliary tract	8
ISHIKAWAHERAKLIO02ER	Endometrium	8
LS411N	Large intestine	8
PATU8902	Pancreas	8
PC3	Prostate	8
SKMEL24	Skin	8
C3A	Liver	8
AN3CA	Endometrium	8
SNGM	Endometrium	8
TE1	Esophagus	8
NCIH1573	Lung	8
HCT116	Large intestine	8
NCIH1568	Lung	8
HPAC	Pancreas	8
HEC151	Endometrium	8
OVMANA	Ovary	8
HCC56	Large intestine	8
HEC1A	Endometrium	8
CAKI2	Kidney	8
CAPAN2	Pancreas	8
NCIH1373	Lung	8
NCIH1048	Lung	8
CAS1	Central nervous system	8
HCC1569	Breast	8
SNU475	Liver	8
LS123	Large intestine	8
NCIH1341	Lung	8
PANC0403	Pancreas	8
MOGGCCM	Central nervous system	8
IM95	Stomach	8
ONCODG1	Ovary	8
NCIH747	Large intestine	8
WM115	Skin	8
DBTRG05MG	Central nervous system	8
EFE184	Endometrium	8
HS695T	Skin	8
KYM1	Soft tissue	8
MORCPR	Lung	8
CORL105	Lung	8
PL45	Pancreas	8
SQ1	Lung	8
TEN	Endometrium	8
T84	Large intestine	8
HCC1395	Breast	8
ZR751	Breast	8
RERFGC1B	Stomach	8
DETROIT562	Upper aerodigestive tract	8
DV90	Lung	8
SW780	Urinary tract	8
KYSE510	Esophagus	8
SKMEL31	Skin	8
NCIH1869	Lung	8
NCIH441	Lung	8
NCIH2085	Lung	8
CORL23	Lung	8
OCUM1	Stomach	8
SNUC2A	Large intestine	8
TE5	Esophagus	8
MKN45	Stomach	8
KP3	Pancreas	8
KNS42	Central nervous system	8
KLE	Endometrium	8
SW1417	Large intestine	8
KMBC2	Urinary tract	8
LC1SQSF	Lung	8
OVSAHO	Ovary	8
VMRCLCD	Lung	8
KP2	Pancreas	8
BT20	Breast	8
RT4	Urinary tract	8
EFM19	Breast	8
KYSE70	Esophagus	8
A253	Salivary gland	8
COLO201	Large intestine	8
SW48	Large intestine	8
SU8686	Pancreas	8
MFE280	Endometrium	8
CAMA1	Breast	8
KURAMOCHI	Ovary	8
COLO678	Large intestine	8
HUPT3	Pancreas	8
HCC1187	Breast	8
T47D	Breast	8
MDAMB415	Breast	8
HSC2	Upper aerodigestive tract	8
KYSE150	Esophagus	8
UACC812	Breast	8
ONS76	Central nervous system	8
KNS62	Lung	8
PANC1005	Pancreas	7.987659
ISTMES2	Pleura	7.889611
NCIH1355	Lung	7.860697
KYSE30	Esophagus	7.858886
22RV1	Prostate	7.847305
MIAPACA2	Pancreas	7.469959
JHOS4	Ovary	7.408363
A204	Soft tissue	7.399833
HCC70	Breast	7.36332
NCIH2286	Lung	7.359588
MALME3M	Skin	7.325411
GCIY	Stomach	7.255416
PK1	Pancreas	7.236271
786O	Kidney	7.178035
T3M10	Lung	7.170651
A2780	Ovary	7.146677
SKLMS1	Soft tissue	7.136584
HT1376	Urinary tract	7.084046
HUPT4	Pancreas	7.0557
PANC0327	Pancreas	6.904092
SW1088	Central nervous system	6.737086
SNU16	Stomach	6.697771
PLCPRF5	Liver	6.669433
HARA	Lung	6.656741
MELHO	Skin	6.552444
RT112	Urinary tract	6.525924
K029AX	Skin	6.444433
EBC1	Lung	6.372372
MCAS	Ovary	6.3241
COLO320	Large intestine	6.295312
PK59	Pancreas	6.190494
HT29	Large intestine	5.884947
TE9	Esophagus	5.855279
WM983B	Skin	5.68912
KCIMOH1	Pancreas	5.619114
TYKNU	Ovary	5.343411
8MGBA	Central nervous system	5.22662
PANC0203	Pancreas	5.197284
NCIH1650	Lung	5.152449
NIHOVCAR3	Ovary	5.117735
OVCAR8	Ovary	5.095931
JHH7	Liver	4.92477
HMCB	Skin	4.767848
MKN74	Stomach	4.689733
HCT15	Large intestine	4.666833
WM793	Skin	4.641666
BXPC3	Pancreas	4.599786
HCC1806	Breast	4.378565
ESS1	Endometrium	4.373962
SCC9	Upper aerodigestive tract	4.287216
MHHES1	Bone	4.274786
A549	Lung	4.227246
HPAFII	Pancreas	4.222833
GCT	Soft tissue	4.213955
C2BBE1	Large intestine	4.099345
KE39	Stomach	4.05606
LU99	Lung	3.926637
VMRCRCW	Kidney	3.895097
KYSE410	Esophagus	3.808475
KYSE520	Esophagus	3.773011
NCIH2030	Lung	3.72418
OE33	Esophagus	3.538352
HDQP1	Breast	3.104604
G361	Skin	3.047757
RL952	Endometrium	3.012983
NCIH2122	Lung	2.934416
NCIH28	Pleura	2.911829
LS513	Large intestine	2.880553
MCF7	Breast	2.845194
NCIH358	Lung	2.83834
ASPC1	Pancreas	2.785628
KYSE450	Esophagus	2.574543
NUGC3	Stomach	2.410753
SCC25	Upper aerodigestive tract	2.398599
SW403	Large intestine	2.379555
LUDLU1	Lung	2.319642
MDAMB468	Breast	2.312559
5637	Urinary tract	2.307768
PC14	Lung	2.149659
L33	Pancreas	2.124577
CAL12T	Lung	1.951666
CAL851	Breast	1.899548
HCC4006	Lung	1.854881
NCIH2444	Lung	1.746528
AZ521	Stomach	1.659918
SCABER	Urinary tract	1.511766
SKMES1	Lung	1.476444
HCC1954	Breast	1.457828
MDAMB453	Breast	1.4379
NCIH322	Lung	1.362128
TE15	Esophagus	1.285878
HCC2935	Lung	1.239924
769P	Kidney	1.057461
MFE319	Endometrium	1.026923
SKOV3	Ovary	0.983712
KYSE180	Esophagus	0.876243
FADU	Upper aerodigestive tract	0.823073
SKCO1	Large intestine	0.71562
KYSE140	Esophagus	0.68893
CAL27	Upper aerodigestive tract	0.688771
CHL1	Skin	0.675993
TE11	Esophagus	0.63775
JHH5	Liver	0.569108
CALU3	Lung	0.494588
MDAMB175VII	Breast	0.468741
NCIH1666	Lung	0.386496
NCIH1648	Lung	0.373409
HCC827	Lung	0.372134
NCIH3255	Lung	0.333763
NCIH2170	Lung	0.300981
TE617T	Soft tissue	0.242928
CCK81	Large intestine	0.240195
SKBR3	Breast	0.196392
AU565	Breast	0.18321
NUGC4	Stomach	0.171543
ZR7530	Breast	0.166593
BT474	Breast	0.116183
NCIN87	Stomach	0.066107

* *Extracted from CCLE database (https://portals.broadinstitute.org/ccle)*.

*IC50 (μM) is half maximal inhibitory concentration (IC50), which is defined as a drug concentration producing absolute 50% inhibition of growth in cell proliferation assay. By definition, this metric relies on the assumption, that at a high concentration of the drug, 100% effect is achieved as all cells die in a proliferation assay*.

### Pathway Analysis Involved in Lapatinib Sensitivity

**Figure 2:**
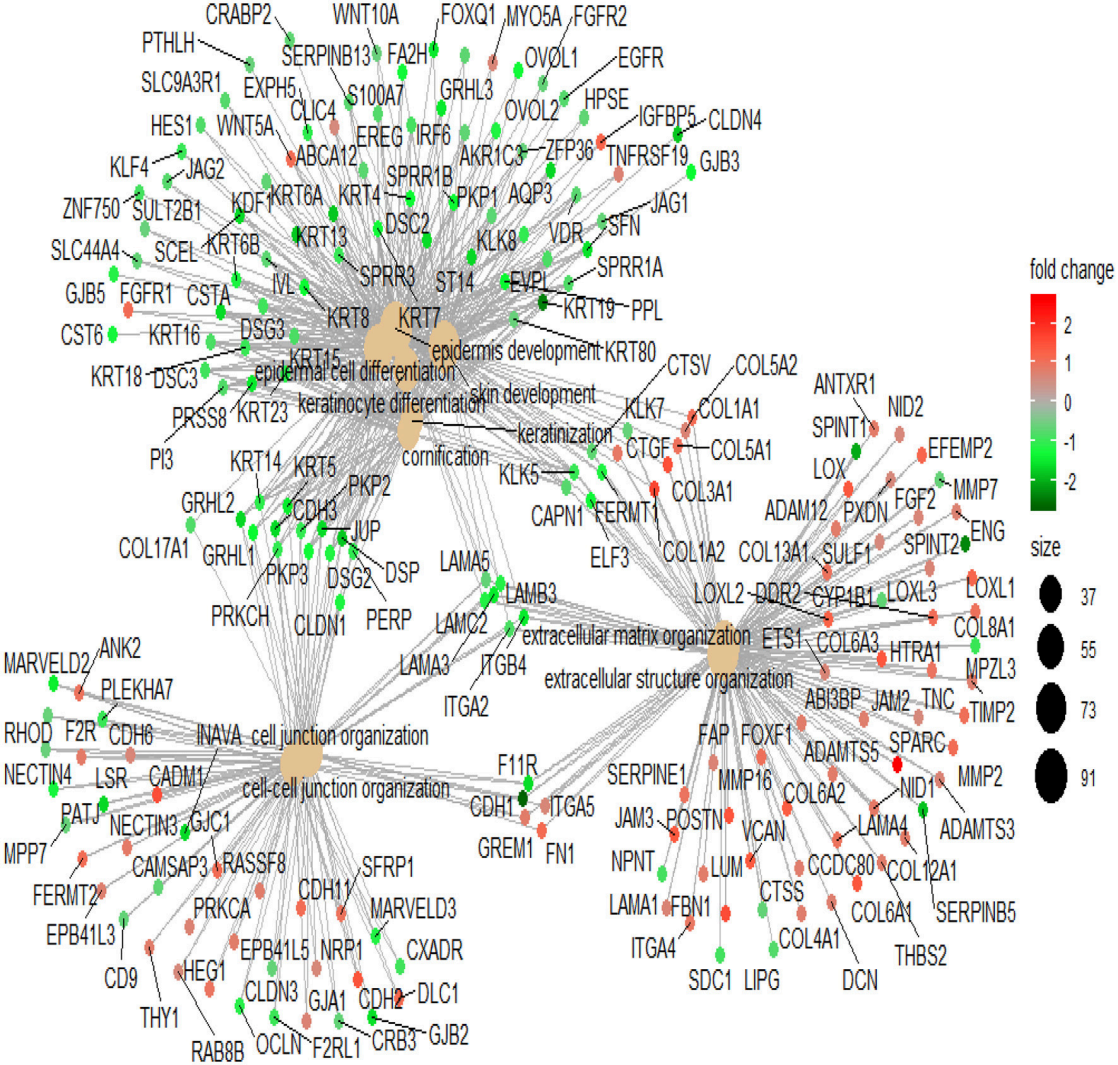
The network of top 10 genes by GO pathway analysis. The large spots in the center of the networks are the gene clusters, and the small spots connected with large spots are the related genes in the pathways. Red spots indicate that the genes are highly expressed in the high_IC50 group. Green spots indicate that the genes are highly expressed in the low_IC50 group. The darker red or green spot are the larger fold-change of differential genes. The black spots with different sizes and numbers on the right side indicate the gene numbers in the gene clusters.

To illustrate the mechanism of Lapatinib resistance, we selected genes with fold-change >1.5 times to perform GO analysis (Table S2). In the top 10 involved pathways, Lapatinib sensitivity was positively associated with cell keratin, epithelial differentiation, and cell-cell junction, while negatively related to signatures of extracellular matrix (Figure 2, *P* < 0.001, *P*. adjust < 0.001).

### Analysis of LncRNAs Involved in Lapatinib Sensitivity

**Figure 3:**
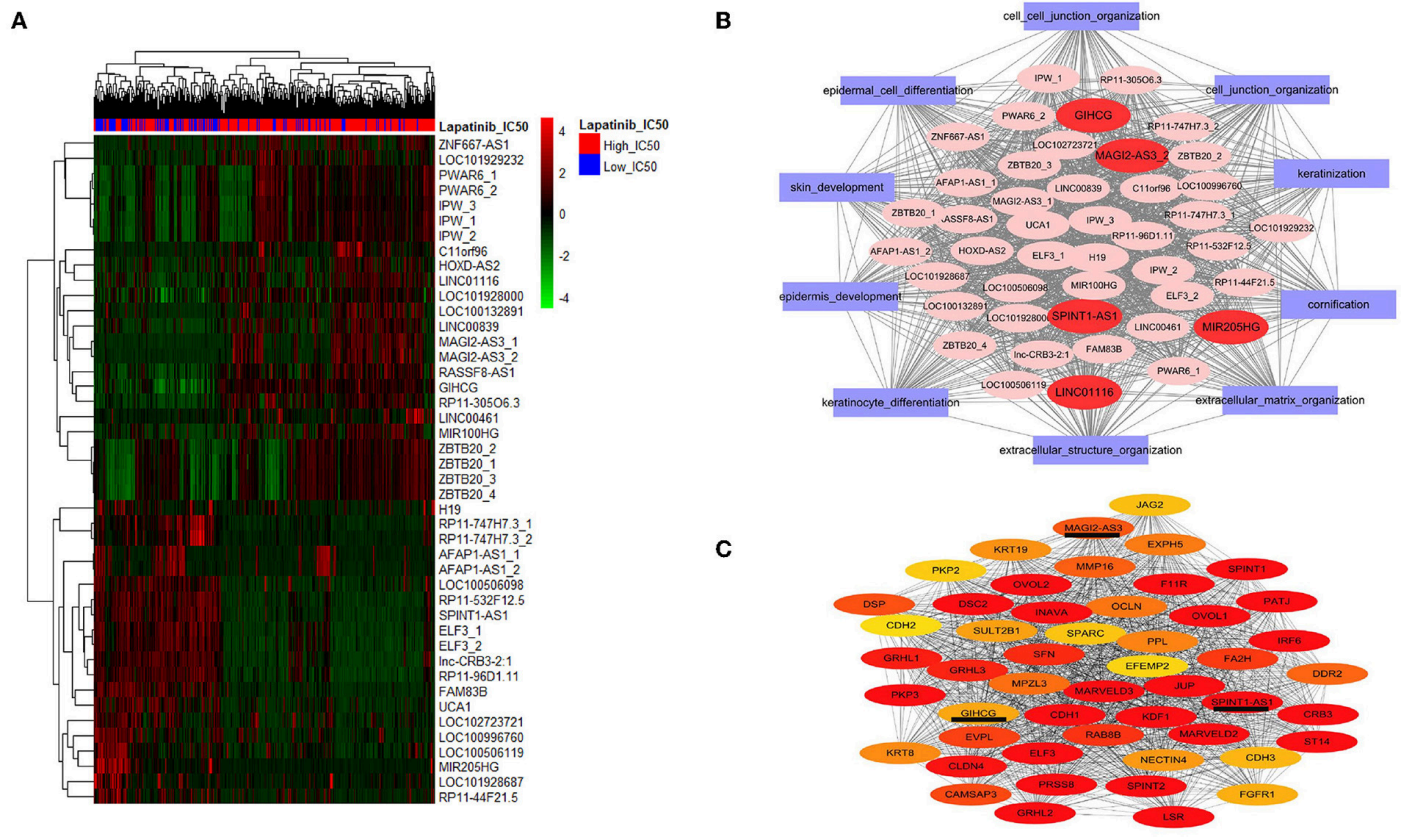
Screening lncRNAs related to Lapatinib sensitivity. **(A)** The heatmap of 44 differentially expressed lncRNAs between high_IC50 group and low_IC50 groups (fold-change >1.5, *P* < 0.05). The red bars on the top present high_IC50 cases, and blue bars represent low_IC50 cases. The numbers of the right side are the names of lncRNAs. The numbers tagged in lncRNAs represent probe codes. **(B)** The co-expression molecular network of the 44 differentially expressed lncRNAs. The red ovals represent five crucial lncRNAs in the network, and the purple rectangles outside indicate the top 10 functional gene sets by GO analysis. **(C)** The co-expression molecular network of the top 50 differentially expressed genes and lncRNAs between the high_IC50 group and the low_IC50 group. In this network, three of differentially expressed molecules are lncRNAs (SPINT1-AS1, MAGI2-AS3, and GIHCG), which are underlined. The colors nodes of the network from red, dark yellow to light yellow indicate gradually weakened correlation to Lapatinib sensitivity.

We further screened the differentially expressed lncRNAs, and 44 lncRNAs were identified between the high_IC50 group and low_IC50 group (Figure 3A and Table 3, fold-change >1.5, *P* < 0.01). Then, we selected genes in the top 10 pathways and 44 differential lncRNAs for the construction of the co-expression network. The enrichment scores of the top 10 pathway genes in every cancer cell lines were calculated and determined by GSVA analysis. Five lncRNAs were highlighted as the hub factors in the top 10 regulating pathways (Figure 3B). The association of the 5 lncRANs with 199 genes in the top 10 pathways was further analyzed, and a molecular network of co-expression was established, which included top 50 key molecules closely associated to Lapatinib sensitivity. Three crucial lncRNAs, GIHCG, SPINT1-AS1, and MAGI2-AS3, still remained in the co-expression network (Figure 3C).

**Table 3:** Differentially expressed lncRNAs between Lapatinib high_IC50 and low_IC50 groups of 420 cancer cell lines (fold-change >1.5, *P* < 0.01).

**Probes**	**Title**	**Symbol**	**Ensemble transcript id version**	**Log FC**	***P*-value**	**Adj. *P*-value**
225381_at	mir-100-let-7a-2 cluster host gene (non-protein coding)	MIR100HG	ENSG00000255248.7	1.339024	4.98E-08	1.48E-05
226546_at	uncharacterized LOC100506844	GIHCG	ENSG00000257698.1	1.19665	1.52E-15	8.13E-12
228564_at	Long intergenic non-protein coding RNA 1116	LINC01116	ENSG00000163364.9	1.122804	4.24E-06	0.000493
227554_at	MAGI2 antisense RNA 3	MAGI2-AS3	ENSG00000234456.7	1.096172	2.73E-07	5.84E-05
1566482_at	NA	RP11-305O6.3	ENSG00000250280.2	0.961776	3.96E-08	1.24E-05
213156_at	Zinc finger and BTB domain containing 20	ZBTB20	ENSG00000259976.3	0.942404	6.68E-06	0.000649
213158_at	Zinc finger and BTB domain containing 20	ZBTB20	ENSG00000259976.3	0.908785	1.6E-05	0.001179
244741_s_at	ZNF667 antisense RNA 1 (head to head)	ZNF667-AS1	ENSG00000166770.10	0.873077	0.000703	0.019471
229480_at	MAGI2 antisense RNA 3	MAGI2-AS3	ENSG00000234456.7	0.870971	4.07E-07	8.05E-05
229493_at	HOXD cluster antisense RNA 2	HOXD-AS2	ENSG00000237380.6	0.795366	2.89E-07	5.94E-05
227082_at	Zinc finger and BTB domain containing 20	ZBTB20	ENSG00000259976.3	0.780225	5.64E-05	0.003174
226587_at	Prader Willi/Angelman region RNA 6	PWAR6	ENSG00000257151.1	0.777959	0.0002	0.008638
242358_at	RASSF8 antisense RNA 1	RASSF8-AS1	ENSG00000246695.7	0.770905	9.02E-08	2.29E-05
236075_s_at	Uncharacterized LOC101928000	LOC101928000	ENSG00000234327.7	0.766575	6.6E-06	0.000649
221974_at	Imprinted in Prader-Willi syndrome (non-protein coding) /// uncharacterized LOC101930404 /// Prader Willi/Angelman region RNA, SNRPN neighbor /// small nucleolar RNA, C/D box 107 /// small nucleolar RNA, C/D box 115–13 /// small nucleolar RNA, C/D box 115–26 /// small nucleolar RNA, C/D box 115–7 /// small nucleolar RNA, C/D box 116–22 /// small nucleolar RNA, C/D box 116–28 /// small nucleolar RNA, C/D box 116–4 /// small nuclear ribonucleoprotein polypeptide N	IPW /// LOC101930404 /// PWARSN /// SNORD107 /// SNORD115-13 /// SNORD115-26 /// SNORD115-7 /// SNORD116-22 /// SNORD116-28 /// SNORD116-4 /// SNRPN	ENSG00000224078.13	0.719911	0.000535	0.016616
227099_s_at	Chromosome 11 open reading frame 96	C11orf96	ENSG00000254409.2	0.686826	0.001963	0.037596
217520_x_at	Uncharacterized LOC101929232 /// PDCD6IP pseudogene 2	PDCD6IPP2	ENSG00000274253.4	0.671638	1.03E-05	0.000862
226591_at	Prader Willi/Angelman region RNA 6	PWAR6	ENSG00000257151.1	0.665136	0.000597	0.018108
233562_at	Long intergenic non-protein coding RNA 839	LINC00839	ENSG00000185904.11	0.644287	0.000226	0.009558
228370_at	Imprinted in Prader-Willi syndrome (non-protein coding) /// uncharacterized LOC101930404 /// Prader Willi/Angelman region RNA, SNRPN neighbor /// small nucleolar RNA, C/D box 107 /// small nucleolar RNA, C/D box 115–13 /// small nucleolar RNA, C/D box 115–26 /// small nucleolar RNA, C/D box 115–7 /// small nucleolar RNA, C/D box 116–22 /// small nucleolar RNA, C/D box 116–28 /// small nucleolar RNA, C/D box 116–4	IPW /// LOC101930404 /// PWARSN /// SNORD107 /// SNORD115-13 /// SNORD115-26 /// SNORD115-7 /// SNORD116-22 /// SNORD116-28 /// SNORD116-4	ENSG00000224078.13	0.63548	0.004004	0.056605
230272_at	Long intergenic non-protein coding RNA 461 /// microRNA 9-2	LINC00461 /// MIR9-2	ENSG00000245526.10	0.633241	0.000333	0.011874
227121_at	Zinc finger and BTB domain containing 20	ZBTB20	ENSG00000259976.3	0.622039	6.47E-05	0.003438
228438_at	Uncharacterized LOC100132891	LOC100132891	ENSG00000235531.9	0.610992	0.00111	0.026335
213447_at	Imprinted in Prader-Willi syndrome (non-protein coding) /// uncharacterized LOC101930404 /// Prader Willi/Angelman region RNA, SNRPN neighbor /// small nucleolar RNA, C/D box 107 /// small nucleolar RNA, C/D box 115–13 /// small nucleolar RNA, C/D box 115–26 /// small nucleolar RNA, C/D box 115–7 /// small nucleolar RNA, C/D box 116–22 /// small nucleolar RNA, C/D box 116–28 /// small nucleolar RNA, C/D box 116–4 /// small nuclear ribonucleoprotein polypeptide N	IPW /// LOC101930404 /// PWARSN /// SNORD107 /// SNORD115-13 /// SNORD115-26 /// SNORD115-7 /// SNORD116-22 /// SNORD116-28 /// SNORD116-4 /// SNRPN	ENSG00000224078.13	0.603999	0.000792	0.021388
238632_at	NA	RP11-44F21.5	ENSG00000260265.1	−0.58771	0.000615	0.018108
224646_x_at	H19, imprinted maternally expressed transcript (non-protein coding) /// microRNA 675	H19 /// MIR675	ENSG00000130600.18	−0.66521	0.008633	0.089285
243729_at	NA	RP11-747H7.3	ENSG00000260711.2	−0.68534	2.63E-09	1.08E-06
1557779_at	Uncharacterized LOC101928687	LOC101928687	ENSG00000231131.6	−0.69525	0.000133	0.006282
229296_at	Uncharacterized LOC100506119	LOC100506119	ENSG00000233901.5	−0.74915	3.12E-05	0.001938
1557094_at	Uncharacterized LOC100996760	LOC100996760	ENSG00000276850.4	−0.80357	4.07E-05	0.002362
223779_at	AFAP1 antisense RNA 1	AFAP1-AS1	ENSG00000272620.1	−0.80513	6.36E-05	0.003434
235921_at	Uncharacterized LOC102723721	LOC102723721	ENSG00000223784.1	−0.81799	9.33E-06	0.000804
1558216_at	AFAP1 antisense RNA 1	AFAP1-AS1	ENSG00000272620.1	−0.84595	0.000286	0.010641
242874_at	NA	RP11-747H7.3	ENSG00000260711.2	−0.92003	9.63E-11	6.43E-08
227985_at	Uncharacterized LOC100506098	LOC100506098	ENSG00000233834.6	−1.04243	7.5E-08	2E-05
236279_at	NA	NA	ENSG00000275234.1	−1.04592	6.12E-10	2.97E-07
232202_at	Family with sequence similarity 83, member B	FAM83B	ENSG00000261116.1	−1.07231	2.29E-10	1.22E-07
238742_x_at	Uncharacterized LOC102724362	SPINT1-AS1	ENSG00000261183.5	−1.10252	6.38E-14	1.7E-10
226755_at	MIR205 host gene (non-protein coding)	MIR205HG	ENSG00000230937.11	−1.11922	1.11E-10	6.61E-08
242354_at	NA	RP11-532F12.5	ENSG00000261183.5	−1.19239	5.99E-13	8.01E-10
229223_at	NA	RP11-96D1.11	ENSG00000262160.1	−1.26926	1.78E-12	1.59E-09
201510_at	E74-like factor 3 (ets domain transcription factor, epithelial-specific)	ELF3	ENSG00000249007.1	−1.54591	1.36E-13	2.42E-10
210827_s_at	E74-like factor 3 (ets domain transcription factor, epithelial-specific)	ELF3	ENSG00000249007.1	−1.63868	8.23E-13	8.79E-10
227919_at	Urothelial cancer associated 1 (non-protein coding)	UCA1	ENSG00000214049.7	−1.65999	9.68E-08	2.35E-05

*log FC, log2 of fold-change. Positive value indicates increased expression in high_IC50 group, and negative value indicates decreased expression in high_IC50 group. NA, Not available*.

### Differential Expressing Analysis of Three LncRNAs Between Epithelial and Non-epithelial Cancer Groups

**Figure 4:**
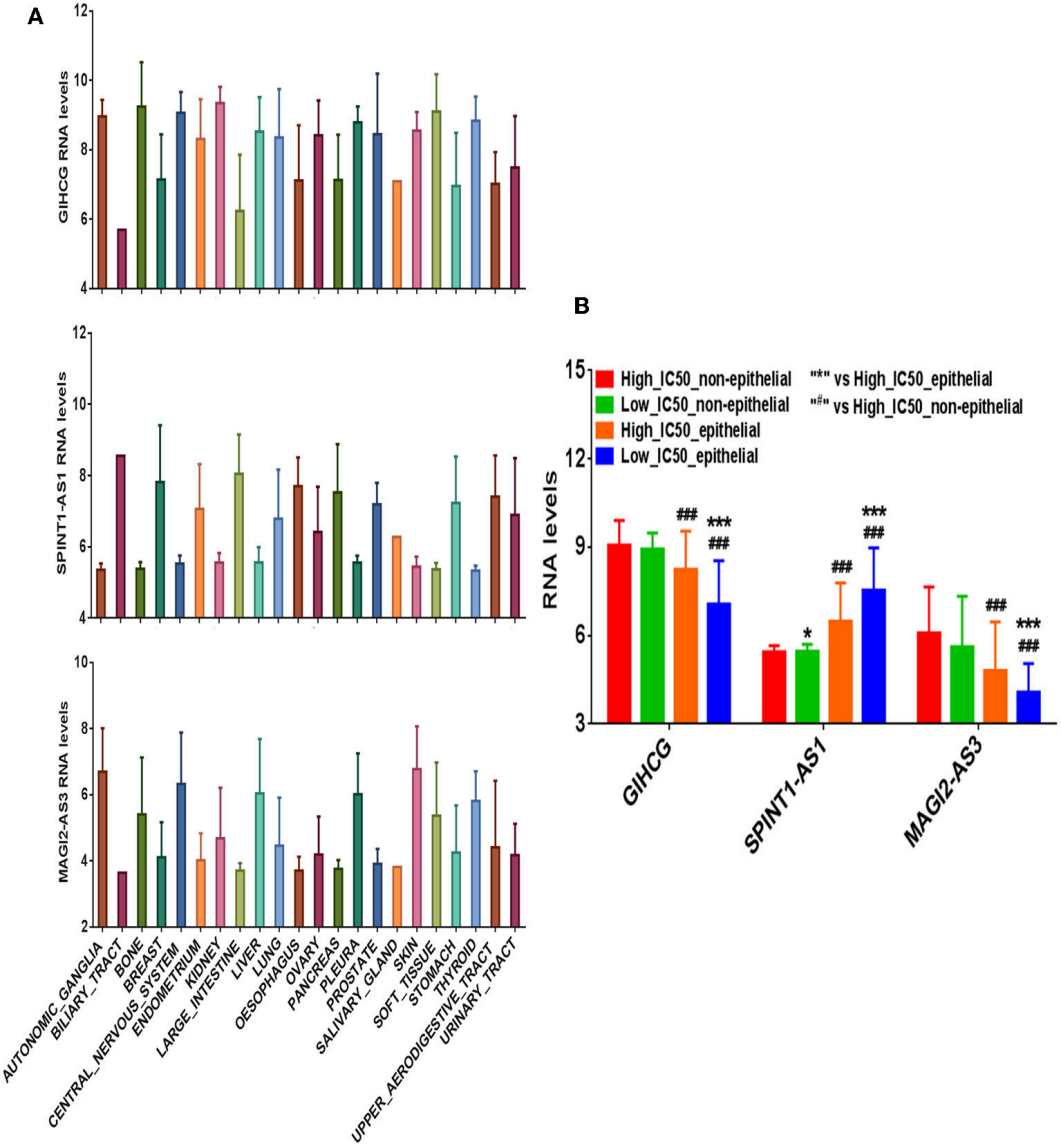
The correlation of expression levels of three crucial lncRNAs and originated sites of cancer cell lines. **(A)** The expression levels of GIHCG, SPINT1-AS1, and MAGI2-AS3 on 22 types of cancer cell lines. **(B)** The bar charts of expression levels of GIHCG, SPINT1-AS1, and MAGI2-AS3 between Lapatinib high_IC50 and low_IC50 groups in epithelial cancer cell lines and non-epithelial cancer cell lines.

We divided the 420 cancer cell lines into epithelium derived group (*n* = 278) and non-epithelium derived group (*n* = 142; including nervous system, bone, cartilage, and pleura). The differential expression levels of the three lncRNAs between the two groups are presented in Figure 4A. In the epithelium-derived group, the differential expression levels of the three lncRNAs between Lapatinib high_IC50 and low_IC50 groups were significantly different (Figure 4B, *P* < 0.05). In the non-epithelium groups, there was no significant difference of the three lncRNAs between Lapatinib high_IC50 and low_IC50 groups. Higher expressing level of SPINT1-AS1 was found in epithelium-derived cancer cells, and higher expressing levels of MAGI2-AS3 and GIHCG were observed in the non-epithelium group.

**Figure 5:**
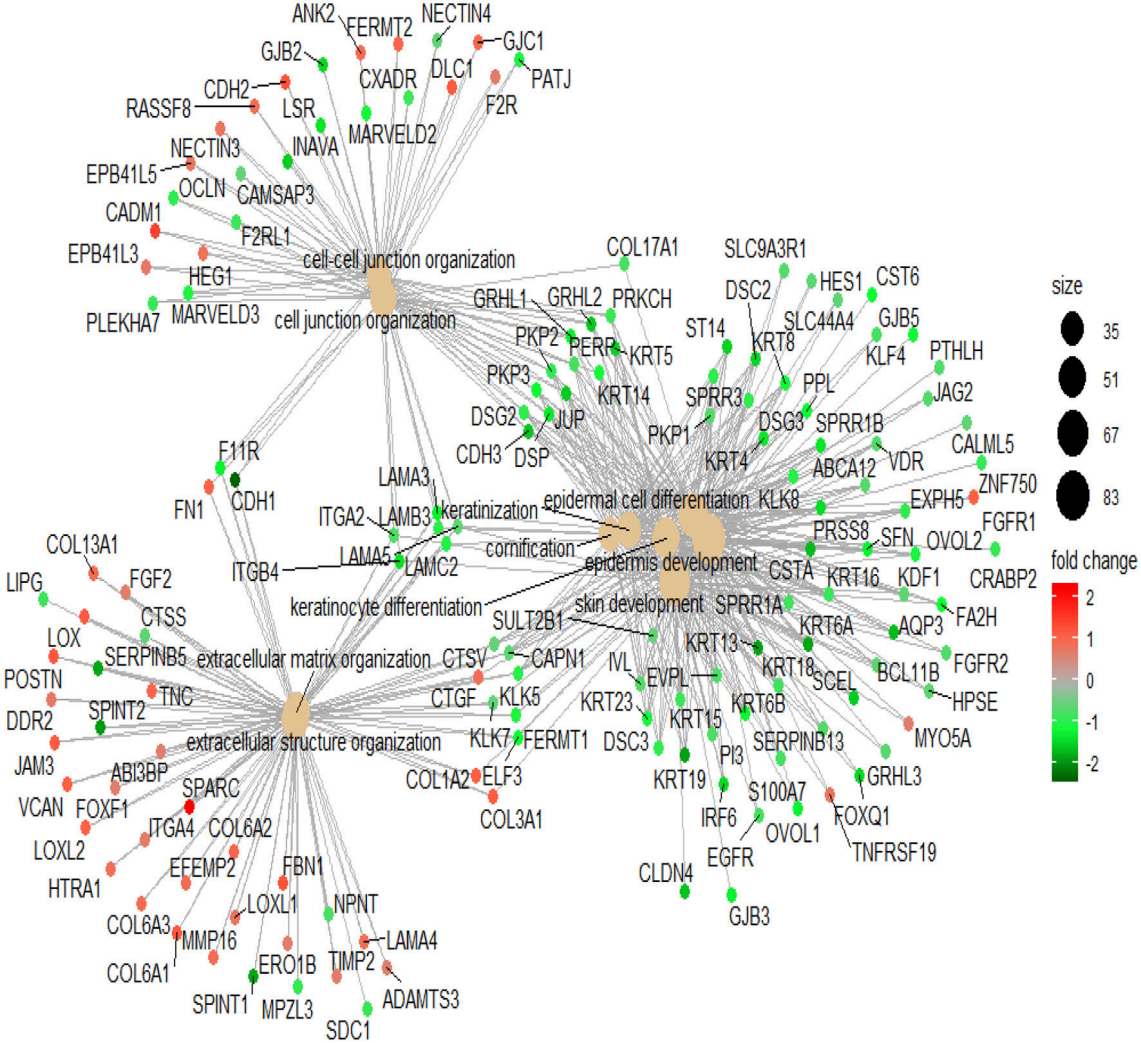
Pathway analysis of Lapatinib sensitivity related genes. The genes in the top 10 pathways with fold-change more than 1.5 are used between Lapatinib high_IC50 and low_IC50 groups. The middle brown dot of each network indicates the name of a gene set, and the small dots surrounding it indicate the genes of the gene set. The red dots represent the up-regulated genes in the high_IC50 group, and the green dots represent the up-regulated genes in the low_IC50 group. The darker red or green spot are the larger fold-change of differential genes. The black spots with different sizes and numbers on the right side indicate the gene numbers in the gene clusters.

Differentially expressed genes (1.5-fold change) between the Lapatinib high_IC50 and low_IC50 groups in epithelial group (Table S3) were utilized to perform GO analysis. Enhanced signatures of cell keratin, epithelial differentiation, and cell-cell junction were observed in Lapatinib low_IC50 group, and decreased signature of extracellular matrix were observed in Lapatinib low_IC50 group (Figure 5, *P* < 0.001, *P*. adjust < 0.001).

### Correlation of LncRNAs SPINT1-AS1, GIHCG, or MAGI2-AS3 and Lapatinib Sensitivity in Epithelial Group

**Figure 6:**
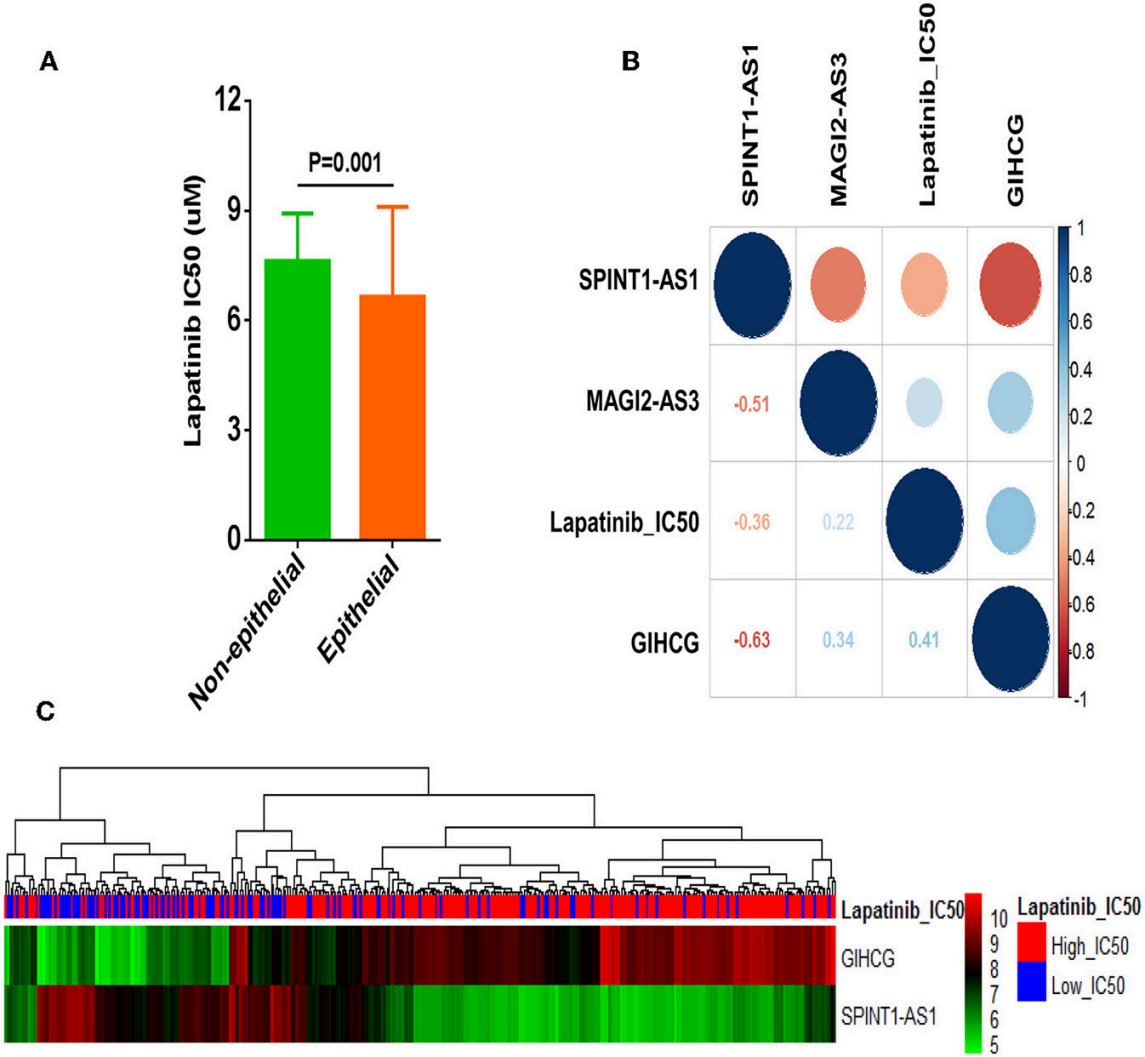
Correlation analysis of three crucial lncRNAs GIHCG, SPINT1-AS1, MAGI2-AS3, and Lapatinib sensitivity in epithelial cancer cells. **(A)** Non-epithelial cancer cells showed higher Lapatinib_IC50 than epithelial cancer cells in the CCLE database. **(B)** Correlation between three lncRNAs and Lapatinib IC50 of epithelial cancer cells. Red circles represent negative correlation, and blue circles represent positive correlation. The number of the lower left grids indicates correlation coefficient between two factors (all *P*-values < 0.001). **(C)** The heatmap presents expression levels of GIHCG and SPINT1-AS1 in Lapatinib high_IC50 and low_IC50 groups of epithelial cancer cell lines.

Correlation analysis revealed that Lapatinib IC50 of the non-epithelial group was higher than that of the epithelial group (Figure 6A). Of the three critical lncRNAs, SPINT1-AS1, and GIHCG were the lncRNAs most correlated to Lapatinib sensitivity (Figure 6B). SPINT1-AS1 and GIHCG were selected as key factors of affecting Lapatinib sensitivity of epithelial cancers. The up-regulation of SPINT1-AS1 was found in low_IC50 group and increased GIHCG was found in high_IC50 group (Figure 6C).

### Validating Study of GIHCG and SPINT1-AS1 on Regulating Lapatinib Sensitivity *in vitro*

**Figure 7:**
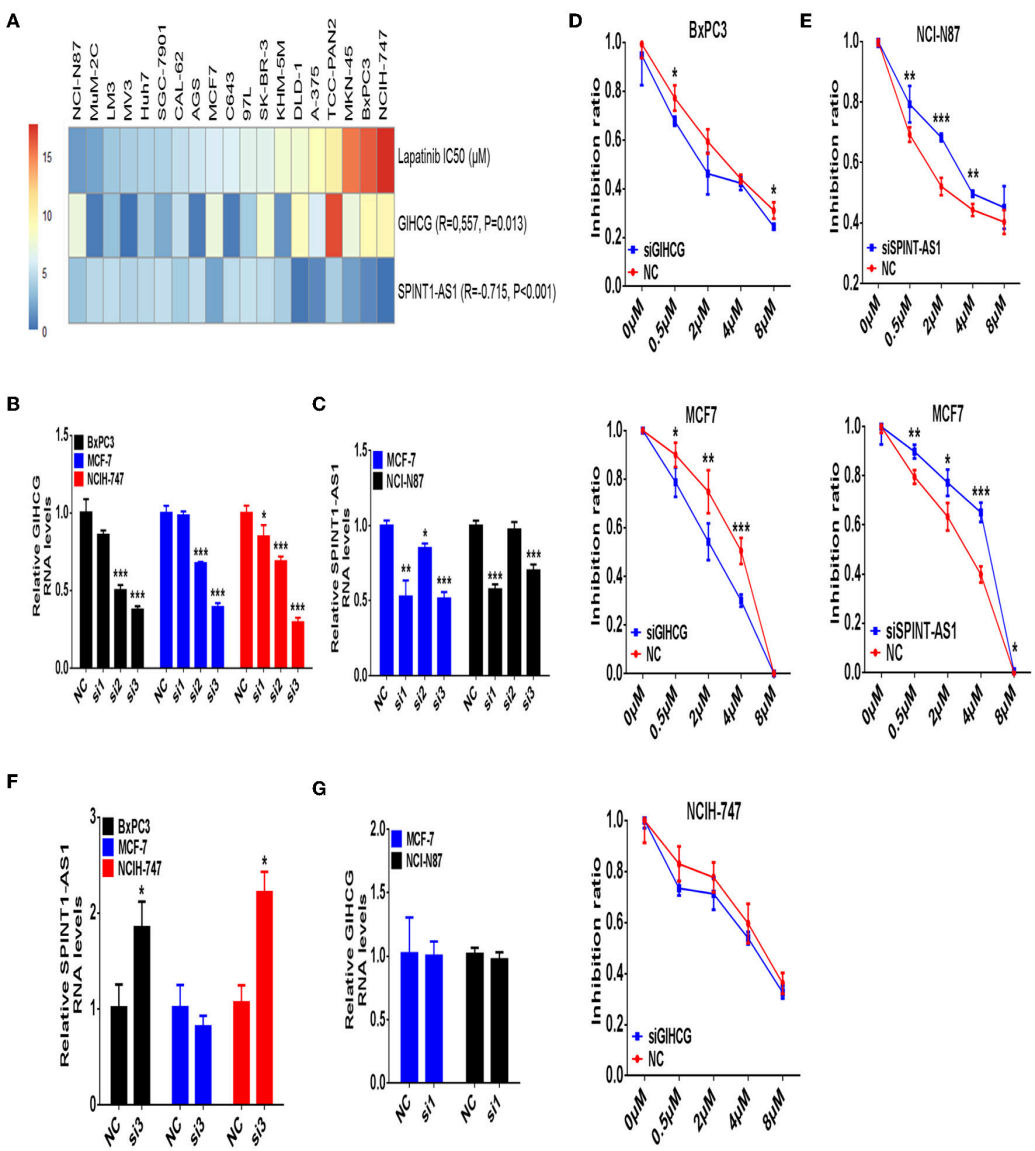
Validating study of lncRNAs GIHCG and SPINT1-AS1 on regulating Lapatinib sensitivity. **(A)** The Lapatinib IC50 and expression levels of GIHCG and SPINT1-AS1 are assayed on 19 cancer cell lines from different types of cancer origin. The expression level of GIHCG is positively related to Lapatinib IC50 (*R* = 0.557, *P* = 0.013), while the expression level of SPINT1-AS1 is negatively related to Lapatinib IC50 (*R* = −0.715, *P* < 0.001). **(B)** Knockdown of GIHCG is performed by siRNA in BxPC3, MCF7, and NCIH-747 cancer cells. **(C)** Knockdown of SPINT1-AS1 is performed by siRNA in NCI-N87 and MCF7 cancer cells. **(D)** Knockdown of GIHCG shows enhancing Lapatinib sensitivity in BxPC3, MCF7, and NCIH-747 cancer cells. **(E)** Knockdown of SPINT1-AS1 shows promoting Lapatinib resistance in NCI-N87 and MCF7 cancer cells. **(F)** Knockdown of GIHCG discloses increased SPINT1-AS1 expression in BxPC3 and NCIH-747 cancer cells. **(G)** Knockdown of SPINT1-AS1 does not increase GIHCG expression in NCI-N87 and MCF7 cancer cells. Experimental group vs. negative control (NC), ^*^P < 0.05, ^**^P < 0.01, ^***^P < 0.001.

In validating experiments, we examined expression levels of GIHCG and SPINT1-AS1 in seven types of cancer cell lines (thyroid cancer, pancreatic cancer, liver cancer, melanoma, gastric cancer, breast cancer, and colorectal cancer) and Lapatinib IC50 of the same cancer cell lines. Correlation analysis showed that higher expression levels of SPINT1-AS1 were significantly associated with lower Lapatinib IC50 (Figure 7A, *R* = −0.715, *P* < 0.001), while higher expression levels of GIHCG were significantly related to higher Lapatinib IC50 (Figure 7A, *R* = 0.557, *P* = 0.013).

The sensitive cancer cell lines of NCI-N87 (gastric cancer) and MCF7 (breast cancer), as well as the resistant cancer cell lines of NCIH-747(colon cancer) and BxPC3 (pancreatic cancer) were selected for a subsequent validating study. After knocking-down expression levels of GIHCG and SPINT1-AS1 by small interfering RNAs, Lapatinib IC50, and inhibitory rate of cancer cells were detected. Among three small interference sequences of GIHCG and SPINT1-AS1 mRNAs, siRNA sequence 3 of GIHCG (Si3, Figure 7B), and siRNA sequence 1 of SPINT1-AS1 (Si1, Figure 7C) were identified as effective siRNAs for further experiments.

Knocking-down of GIHCG could significantly enhance the sensitivity to Lapatinib in MCF7 and BxPC3 cancer cell lines (Figure 7D), while down-regulation of SPINT1-AS1 could promote resistance to Lapatinib in NCI-N87 and MCF7 cancer cell lines (Figure 7E). To clarify whether there is a mutual regulatory relationship between GIHCG and SPINT1-AS1, we detected the expression level of SPINT1-AS1 after GIHCG knockdown and vice versa. As shown in Figures 7F,**G**, suppression of GIHCG in Lapatinib resistant cancer cell lines NCIH-747 and BxPC3 could induce up-regulation of SPINT1-AS1 (*P* < 0.05), while knockdown of SPINT1-AS1 did not change the expression level of GIHCG (*P* > 0.05).

## Discussion

LncRNA is an important regulatory molecule in drug resistance during chemotherapy or gene targeted therapy (Li et al., 2016; Dong et al., 2018; Wu et al., 2018; Zhou et al., 2018). In this study, we analyzed Lapatinib sensitivity to EGFR and ERBB2 targeted therapy pan-cancer cell line wide. We noticed that Lapatinib sensitivity was not only positively correlated to the activation of EGFR and ERBB2 signaling pathways, but also positively associated to cell keratin, epithelial differentiation, and cell-cell junction. The Lapatinib sensitivity of cancer cell lines was negatively associated to extracellular matrix signature. By screening differentially expressed lncRNAs and establishing co-expression network between Lapatinib high_IC50 and low_IC50 groups, three key lncRNAs, SPINT1-AS1, GIHCG, and MAGI2-AS3, were found. Of those, GIHCG and SPINT1-AS1 were only differentially expressed in epithelial derived cancers. SPINT1-AS1 was negatively related to Lapatinib IC50, whereas GIHCG was positively associated to Lapatinib IC50. By siRNAs treatment, downregulation of SPINTA-AS1 could promote Lapatinib resistance, while downregulation of GIHCG promoted Lapatinib sensitivity. The combination of bioinformatical approach and experimental study confirmed that lncRNAs were involved in regulating sensitivity to Lapatinib targeted therapy.

PI3K/Akt, Ras/Raf/MEK/ERK1/2, and PLCγ pathways are downstream pathways of EGFR and ERBB2 and play important roles in cell proliferation and survival of multiple cancers (Roskoski, 2014). The expression levels of EGFR and ERBB2 are positively correlated to Lapatinib sensitivity (Rusnak et al., 2007; Xiang et al., 2018). Trastuzumab (Herceptin) is a molecular targeted drug of ERBB2-positive metastatic/advanced breast cancer and gastric cancer (Bang et al., 2010; Loibl and Gianni, 2017). Lapatinib is a small molecule chemical, which proved effective for ERBB2-positive advanced or metastatic breast cancer when combined with capecitabine after previous treatment with anthracyclines, paclitaxel, or trastuzumab (Geyer et al., 2006). In gastric cancer, treatment with Lapatinib plus capecitabine and oxaliplatin also revealed anti-cancer effects on HER2-amplified gastroesophageal adenocarcinoma, especially in Asian and younger patients (Hecht et al., 2016). LncRNAs emerged as one of the new resistance mechanisms to chemotherapy or molecule targeted therapy. By bioinformatics analysis, Lapatinib sensitive cancer cells exhibited enrichment of genes related to cell keratin, epithelial differentiation, and cell-cell junction. The ERBB family plays an important role in regulating cell differentiation (Pellat et al., 2017). We noticed that Lapatinib sensitivity is positively correlated to ERBB pathway activation. It means that cancer cells sensitive to Lapatinib drug often showed enrichment of cell differentiation-related genes, while Lapatinib-resistant cancer cells are often accompanied by enrichment of extracellular matrix pathway (D'Amato et al., 2015; Khan et al., 2016; Lin et al., 2017; Watson et al., 2018). Furthermore, increases of extracellular matrix could further induce epithelial-mesenchymal transition of cancer cells (Tzanakakis et al., 2018).

Although the role of lncRNAs in cancer progression and Lapatinib resistance have been reported in other studies (Russell et al., 2015; Li et al., 2016; Liang et al., 2018; Ma et al., 2018), this is the first study that proved that lncRNAs GIHCG and SPINT1-AS1 are involved in regulating therapeutic sensitivity to Lapatinib. Based on pan-cancer cell lines analysis, Lapatinib IC50 is significantly different between non-epithelial cancer cell lines, and epithelial cancer cell Lines. As the inhibitor of miR-200b/200a/429, LncRNA GIHCG was shown effectively promoting the progression of liver cancer through inducing methylation of miR-200b/200a/429 promoter (Sui et al., 2016). GIHCG is also involved in promoting cancer proliferation and migration in tongue and renal cancers (D'Aniello et al., 2018; Ma et al., 2018). However, there is no study $om whether or not GIHCG could regulate Lapatinib drug sensitivity in cancers. LncRNA SPINT1-AS1 is a Kunitz type 1 antisense RNA1, belonging to serine peptidase inhibitor. An increased expression of SPINT1-AS1 has been observed in colorectal cancer (Li C. et al., 2018). It is also the first time that lncRNA SPINT1-AS1 has been found regulating Lapatinib drug sensitivity on multiple cancer cells. In validating experiments, the knockdown of SPINT1-AS1 did not result in the up-regulation of GIHCG. We speculated that GIHCG may regulate SPINT1-AS1 expression through regulating promoter methylation or by manner of competitive endogenous RNA (ceRNA) (Zhang G. et al., 2018; Zhang L. et al., 2018). However, the mutual regulatory mechanisms of lncRNA GIHCG and SPINT1-AS1 remain to be studied in the future.

## Conclusion

In conclusion, the current study proposed a group of lncRNAs related to Lapatinib sensitivity based on pan-cancer cell lines analysis. By subsequent experimental study, lncRNAs GIHCG and SPINT1-AS1 were firstly identified as crucial lncRNAs in regulating Lapatinib resistance or sensitivity in epithelium-derived cancer cell lines. SPINT1-AS1 is a Lapatinib sensitivity predictor, while GIHCG is a predictive molecule for Lapatinib resistance.

## Ethics Statement

The protocols used in this study were approved by Rui Jin Hospital Ethics Review Boards. Written informed consents were obtained from all human material donors in accordance with the Declaration of Helsinki. Animals were used according to the protocols approved by Rui Jin Hospital Animal Care and Use Committee.

## Author Contributions

KL and YY conceived and designed the experiments. ZX, ShS, ZZ, JG, and QL performed the experiments. ZX, ZZ, SaS, WS, YY, and KL analyzed the data. ZX, ShS, ZZ, SaS, WS, YY, and KL contributed reagents, materials, and analysis tools. ZX, YY, and KL wrote the paper.

### Conflict of Interest Statement

The authors declare that the research was conducted in the absence of any commercial or financial relationships that could be construed as a potential conflict of interest.
